# Increased Derived Time in Range Is Associated with Reduced Risk of Major Adverse Cardiovascular Events, Severe Hypoglycemia, and Microvascular Events in Type 2 Diabetes: A Post Hoc Analysis of DEVOTE

**DOI:** 10.1089/dia.2022.0447

**Published:** 2023-05-29

**Authors:** Richard M. Bergenstal, Elise Hachmann-Nielsen, Kajsa Kvist, Anne L. Peters, Jens Magelund Tarp, John B. Buse

**Affiliations:** ^1^ International Diabetes Center, HealthPartners Institute, 3800 Park Nicollet Blvd, Minneapolis, Minnesota, USA.; ^2^ Clinical Drug Development, Novo Nordisk A/S, Søborg, Denmark.; ^3^ Data Science, Novo Nordisk A/S, Søborg, Denmark.; ^4^ Keck School of Medicine of the University of Southern California, Los Angeles, California, USA.; ^5^ Department of Medicine, University of North Carolina School of Medicine, Chapel Hill, North Carolina, USA.

**Keywords:** Cardiovascular disease, Continuous glucose monitoring, Diabetes complications, Diabetic retinopathy, Hypoglycemia

## Abstract

Time spent in glycemic target range (time in range [TIR]; plasma glucose 70–180 mg/dL [3.9–10.0 mmol/L]) as a surrogate endpoint for long-term diabetes-related outcomes requires validation. This post hoc analysis investigated the association between TIR, derived from 8-point glucose profiles (derived TIR [dTIR]) at 12 months, and time to cardiovascular or severe hypoglycemic episodes in people with type 2 diabetes in the DEVOTE trial. At 12 months, dTIR was significantly negatively associated with time to first major adverse cardiovascular event (*P* = 0.0087), severe hypoglycemic episode (*P* < 0.0001), or microvascular event (*P* = 0.024). A nonsignificant trend was seen toward association between 12-month hemoglobin A1c (HbA1c) and these outcomes, but this was no longer seen after addition of dTIR to the model. The results support targeting TIR >70% and suggest dTIR could be used in addition to, or in some instances in place of, HbA1c as a clinical biomarker.

**Trial registration details:**

ClinicalTrials.gov, NCT01959529

## Introduction

Hemoglobin A1c (HbA1c) has long been regarded as the “gold standard” for assessing glycemic control. More recently, measurement of the percentage of time people with diabetes spend in target glycemic range (typically 70–180 mg/dL [3.9–10.0 mmol/L]), otherwise known as time in range (TIR), has been acknowledged as an important glucose metric.^1^ TIR, assessed by continuous glucose monitoring (CGM),^2,3^ may generate additional information valuable to the optimization of glucose management. TIR has been shown to correlate with reduced risks of adverse macrovascular outcomes (or surrogates of risk)^4–8^ including reduced cardiovascular and all-cause mortality,^6^ reduced risk of adverse microvascular outcomes,^1,7,9,10^ and reduced cancer mortality.^11^

The current recommended target for most people with diabetes is to achieve >70% of daily readings (or ∼17 h/day) within target glycemic range.^12^ A lower percentage target may be considered in older individuals and/or those at higher risk of severe hypoglycemia due to age, duration of diabetes, duration of insulin therapy, and greater prevalence of hypoglycemia unawareness, in whom the risks of treatment may exceed the benefits of lower glucose levels.^12^ For such patients, minimizing time below target range might be considered the priority.

A TIR (70–180 mg/dL [3.9–10.0 mmol/L]) of 70% for a population corresponds to HbA1c ∼7.0%, a TIR of 50% corresponds to HbA1c ∼8.0%, and every 10% increase in TIR is associated with an ∼0.5% improvement in HbA1c.^12,13^ In addition to measurement using CGM, derived TIR (dTIR) may be estimated from self-measured blood glucose (SMBG) profiles.

A post hoc analysis from the Diabetes Control and Complications Trial (DCCT) reported a negative relationship between dTIR from 7-point glucose profiles obtained (over the course of a day) every 3 months and the development or progression of retinopathy, or development of microalbuminuria, subsequent to this, in people with type 1 diabetes.^1^ The hazard rate for retinopathy progression increased by 64% (95% confidence interval [CI], 51–78) for each 10 percentage points lower dTIR (*P* < 0.001). This suggests that a simple dTIR metric derived from a self-monitored glucose profile may have clinical value. However, more data are needed to further validate such a dTIR as a surrogate endpoint for diabetes-related complications, particularly long-term complications.

The DEVOTE cardiovascular outcomes trial comparing insulin degludec (degludec) versus insulin glargine 100 U/mL (glargine U100) in a type 2 diabetes (T2D) population included many participants with 8-point glucose profiles and adjudicated events. This post hoc analysis of the DEVOTE trial investigated the association between TIR derived from 8-point SMBG profiles and time to first major adverse cardiovascular event (MACE), severe hypoglycemic episode, or microvascular event.

## Materials and Methods

The study design of the DEVOTE trial (NCT01959529) was described previously (Supplementary Fig. S1).^14,15^ The protocol was approved by the institutional review board or independent ethics committee at each site.^15^ In brief, 7637 people with T2D were randomized to degludec (*n* = 3818) or glargine U100 (*n* = 3819) once daily, with or without standard of care. Most participants (85.2%) had established cardiovascular (CV) disease, chronic kidney disease, or both, and were, therefore, at high risk for CV events. Eligible participants were being treated with ≥1 oral or injectable antihyperglycemic agent and had HbA1c ≥7% (or HbA1c <7% if treated with ≥20 U/day of basal insulin).^14,15^ Participants could continue their pretrial antihyperglycemic therapy but basal or premix insulins were discontinued.^14,15^

The primary composite outcome was first occurrence of an adjudicated MACE (death from CV causes, nonfatal myocardial infarction, or nonfatal stroke) with a prespecified noninferiority margin of 1.3. Adjudicated severe hypoglycemia, as defined by the American Diabetes Association and The Endocrine Society as an episode requiring the assistance of another person to administer carbohydrate or glucagon, or to take other corrective actions,^16^ was the prespecified multiplicity-adjusted secondary outcome. The DEVOTE trial had a median observation time of 1.99 years (minimum 0.00 years; maximum 2.75 years). Any differences between the 5774 participants in this analysis and the full trial population were assumed to be negligible.

This post hoc analysis used 8-point SMBG profiles obtained at 12 months that had ≥6 points available. Therefore, although referred to as 8-point SMBG data, in some cases, data were derived from less than eight timepoints. Data were pooled from both treatment arms. Individual dTIR at 12 months was defined as the proportion of glucose values from the 8-point SMBG profiles within target range (70–180 mg/dL [3.9–10.0 mmol/L]). MACE, severe hypoglycemic episodes, and microvascular events (diagnosis of retinopathy or chronic kidney disease [MedDRA defined]) throughout the DEVOTE trial were used.

MACE and severe hypoglycemic episodes were externally adjudicated in the original analysis, whereas microvascular events were derived from safety reports.^14,15^ A Cox model was used to estimate the association between dTIR and time to first MACE, severe hypoglycemic episode, or microvascular event. Hazard ratios (HRs) were estimated for participants with dTIR >50 to ≤70% versus dTIR ≤50%, dTIR >70% versus dTIR ≤50%, dTIR >50% versus dTIR ≤50%, and dTIR >70% versus dTIR ≤70%.

Associations between HbA1c at baseline or at 12 months and time to first MACE, severe hypoglycemic episode, or microvascular event were also measured to compare the predictive value of HbA1c to dTIR for these events. To further elucidate the association between SMBG and severe hypoglycemic episodes, a Cox model was fitted with derived time below range (dTBR).

## Results

Supplementary Figure S2 shows the 8-point SMBG profiles pooled from both treatment arms in the DEVOTE trial at 12 months, 24 months, and end of treatment. Of the 7637 people with T2D randomized in the DEVOTE trial, 5774 had 8-point SMBG profiles with ≥6 points available at 12 months (5268 with eight measurements, 376 with seven measurements, and 130 with six measurements). Baseline characteristics for participants are given in Supplementary Table S1.

At 12 months, dTIR was >70% for 65% of participants included in the post hoc analysis, and mean (± standard deviation) dTIR was 74 (±24)%. Of the 681 MACEs recorded in the DEVOTE trial,^15^ 370 were in participants with 8-point SMBG profiles. Of the 752 severe hypoglycemic episodes in the DEVOTE trial,^15^ 314 occurred in participants with 8-point SMBG profiles.

**FIG. 1.**
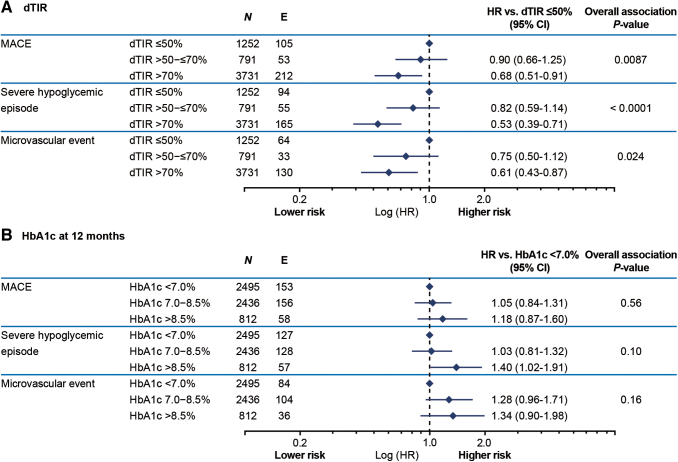
Association between time to first MACE, severe hypoglycemic episode, or microvascular event and **(A)** dTIR or **(B)** HbA1c, at 12 months. CI, confidence interval; dTIR, derived time in range; E, number of events; HbA1c, hemoglobin A1c; HR, hazard ratio; MACE, major adverse cardiovascular event; *N*, number of participants with dTIR or HbA1c.

Figure 1A shows the association between dTIR at 12 months and time to first MACE, severe hypoglycemic episode, or microvascular event. At 12 months, dTIR was significantly negatively associated with time to first MACE (*P* = 0.0087), severe hypoglycemic episode (*P* < 0.0001), or microvascular event (*P* = 0.024), regardless of cutoff value (dTIR >50 to ≤70% or dTIR >70%) compared with dTIR ≤50%. Furthermore, dTIR was significantly negatively associated with time to first MACE, severe hypoglycemic episode, or microvascular event for dTIR >50% compared with dTIR ≤50% (*P* = 0.037, *P* < 0.001 and *P* = 0.015, respectively), and for dTIR >70% compared with dTIR ≤70% (*P* = 0.0025, *P* < 0.00001 and *P* = 0.020, respectively; Supplementary Fig. S3).

**FIG. 2.**
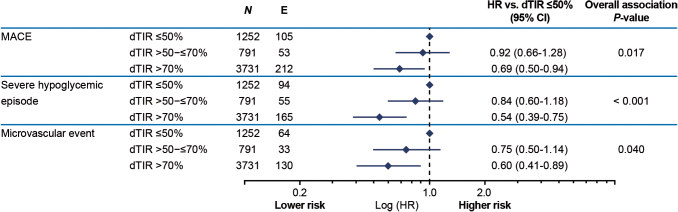
Association between dTIR at 12 months and time to first MACE, severe hypoglycemic episode, or microvascular event, after addition of HbA1c to the model. CI, confidence interval; dTIR, derived time in range; E, events; HR, hazard ratio; MACE, major adverse cardiovascular event; *N*, number of participants with dTIR and HbA1c at 12 months.

Associations and HRs were consistent when analyses were adjusted for baseline age, gender, diabetes duration, HbA1c, insulin use, and CV risk. The negative association between dTIR at 12 months and time to first MACE, severe hypoglycemic episode, or microvascular event remained statistically significant after adding HbA1c to the model (*P* = 0.017, *P* < 0.001 and *P* = 0.040, respectively; Fig. 2).

A significant positive association was seen between baseline HbA1c and time to first MACE or microvascular event (MACE: HR 1.13 [95% CI, 1.07–1.20], *P* < 0.0001; microvascular event: HR 1.09 [95% CI, 1.01–1.17], *P* = 0.034). A trend was seen toward an association between HbA1c at 12 months and time to first MACE, severe hypoglycemia episode, or microvascular event, but was not statistically significant (*P* = 0.56, *P* = 0.10 and *P* = 0.16, respectively; Fig. 1B). The trend toward an association was no longer seen when dTIR was added to the model (*P* = 0.95, *P* = 0.52 and *P* = 0.56, respectively; Supplementary Fig. S4).

Association between dTIR at 12 months and baseline HbA1c was considered clinically insignificant (correlation coefficient −0.19). A more noteworthy association was seen, however, between dTIR at 12 months and HbA1c at 12 months (correlation coefficient −0.43).

**FIG. 3.**
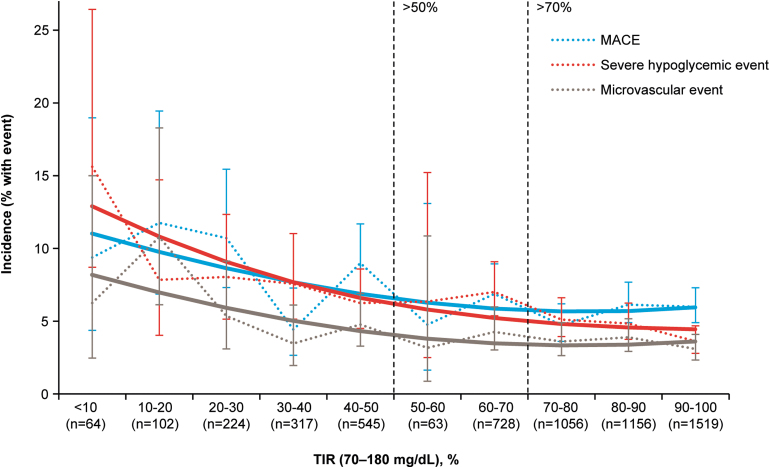
Relationship between dTIR at 12 months and incidence of MACEs, severe hypoglycemic episodes, or microvascular events. Dotted lines represent incidence of outcomes for each TIR percentile. Error bars represent the 95% CI for the plotted incidence. Solid lines represent smoothing of data (to aid visual assessment) for a MACE (blue line), severe hypoglycemic event (red line), and microvascular event (gray line). CI, confidence interval; dTIR, derived time in range; MACE, major adverse cardiovascular event; *n*, number of participants with dTIR; TIR, time in range.

Participants with a greater percentage of dTIR at 12 months had lower observed incidence of MACE, severe hypoglycemia episodes, or microvascular events than those with a lower percentage of dTIR (Fig. 3). For dTIR >70%, the incidence of MACE, severe hypoglycemia episodes, and microvascular events was 5.7%, 4.4%, and 3.5%, respectively. For dTIR >50 to ≤70%, the incidence of MACE, severe hypoglycemic episodes, and microvascular events was 6.7%, 7.0%, and 4.2%, respectively, whereas for dTIR ≤50%, the incidence was 8.4%, 7.5%, and 5.1%, respectively. Risk of MACE was reduced by 6% (HR 0.94; 95% CI, 0.9–0.98; *P* < 0.05), and risk of severe hypoglycemia was reduced by 10% (HR 0.9; 95% CI, 0.86–0.93; *P* < 0.05], for each 10 percentage points greater dTIR.

Reduced dTBR was also associated with a lower risk of severe hypoglycemia, the risk increasing by 32% (HR 1.32; 95% CI, 1.18–1.47; *P* < 0.0001], for each 10 percentage points greater dTBR. dTBR and dTIR were inversely correlated at 12 months (correlation coefficient −0.17). The reduction in risk of microvascular events was not calculated since these were derived from safety reports and not independently adjudicated.

## Discussion

In this post hoc analysis of people with T2D from the DEVOTE trial, dTIR at 12 months based on 8-point SMBG profile data was significantly negatively associated with time to first MACE, severe hypoglycemic episode, or microvascular event. This association remained statistically significant when HbA1c was included in the model. This implies that the metric of TIR or dTIR is not only a helpful diabetes management tool independent of HbA1c but may also be an equally good risk indicator for acute and chronic diabetes complications.

Individuals with a greater dTIR also had a lower observed incidence of MACE and severe hypoglycemia than those with less dTIR. Predictably, reduced dTBR was associated with a lower risk of severe hypoglycemia, also evidenced by the inverse correlation between dTBR and dTIR.

Validation and acceptance of TIR as an endpoint and glycemic metric could be beneficial to people with diabetes, particularly in the current pandemic/postpandemic era, as reliance on “cloud”-based data, remote monitoring, and telemedicine is needed and expected to increase. TIR measurements can be taken and saved to the “cloud” and do not necessarily require direct contact with health care professionals. To our knowledge, this is the first demonstrated association using clinical trial data of dTIR with clinically meaningful outcomes in people with T2D.

These findings are aligned with previously reported data from the DCCT,^1^ as well as other studies investigating the association between time spent in glycemic target range and risks for adverse macrovascular^4–8^ and microvascular outcomes,^1,6,7,9,10,17,18^ all supporting the potentially beneficial effect of greater TIR on reducing the risk of long-term diabetes complications.

The way dTIR data were calculated for this analysis, i.e., derived from SMBG data, is a limitation, as SMBG data are not as sensitive as CGM data. However, given the lack of data on CGM-based TIR and long-term complications in CV outcomes trials, dTIR may be considered a proxy for CGM-based TIR, and may provide clinical insight into the link between glycemic fluctuations and diabetes-related complications.

It is also worth noting that, as dTIR was estimated from six to eight SMBG measurements over the course of 1 day at the 12-month timepoint, it is possible that events may have occurred before or after this point. An outcome occurring in the first 12 months could have affected adherence to medication or triggered changes to lifestyle that could have, in turn, affected the 12-month dTIR measurement. Finally, although a relationship was shown between dTIR and incidence of MACE, severe hypoglycemia, or microvascular events, it is worth noting that low number of events in dTIR percentiles <50% resulted in relatively large sampling errors.

A trend was seen toward an association between HbA1c at 12 months and time to first MACE, severe hypoglycemic episode, or microvascular event, but this was not statistically significant, and the trend was no longer seen when dTIR was added to the model. This suggests that HbA1c does not strengthen the association of dTIR with time to first MACE, severe hypoglycemic episode, or microvascular event.

## Conclusions

The results support the recommendation of striving for TIR >70% as a primary target and validate the clinical value of a secondary target of >50% TIR. Furthermore, they suggest that dTIR could be used in addition to, or in some instances in place of, HbA1c as a clinical biomarker.

## Supplementary Material

Supplemental data

Supplemental data

Supplemental data

Supplemental data

Supplemental data
