# Supplementary material for: Increased Derived Time in Range Is Associated with Reduced Risk of Major Adverse Cardiovascular Events, Severe Hypoglycemia, and Microvascular Events in Type 2 Diabetes: A Post Hoc Analysis of DEVOTE
Source: Diabetes Technol Ther. 2023 May 29;25(6):378–83. doi: 10.1089/dia.2022.0447 (PMC10398723; doi:10.1089/dia.2022.0447)
Supplement: Supplemental data [file Suppl_FigureS2.docx]

**Figure S2.** Combined 8-point SMBG profiles in the DEVOTE trial.


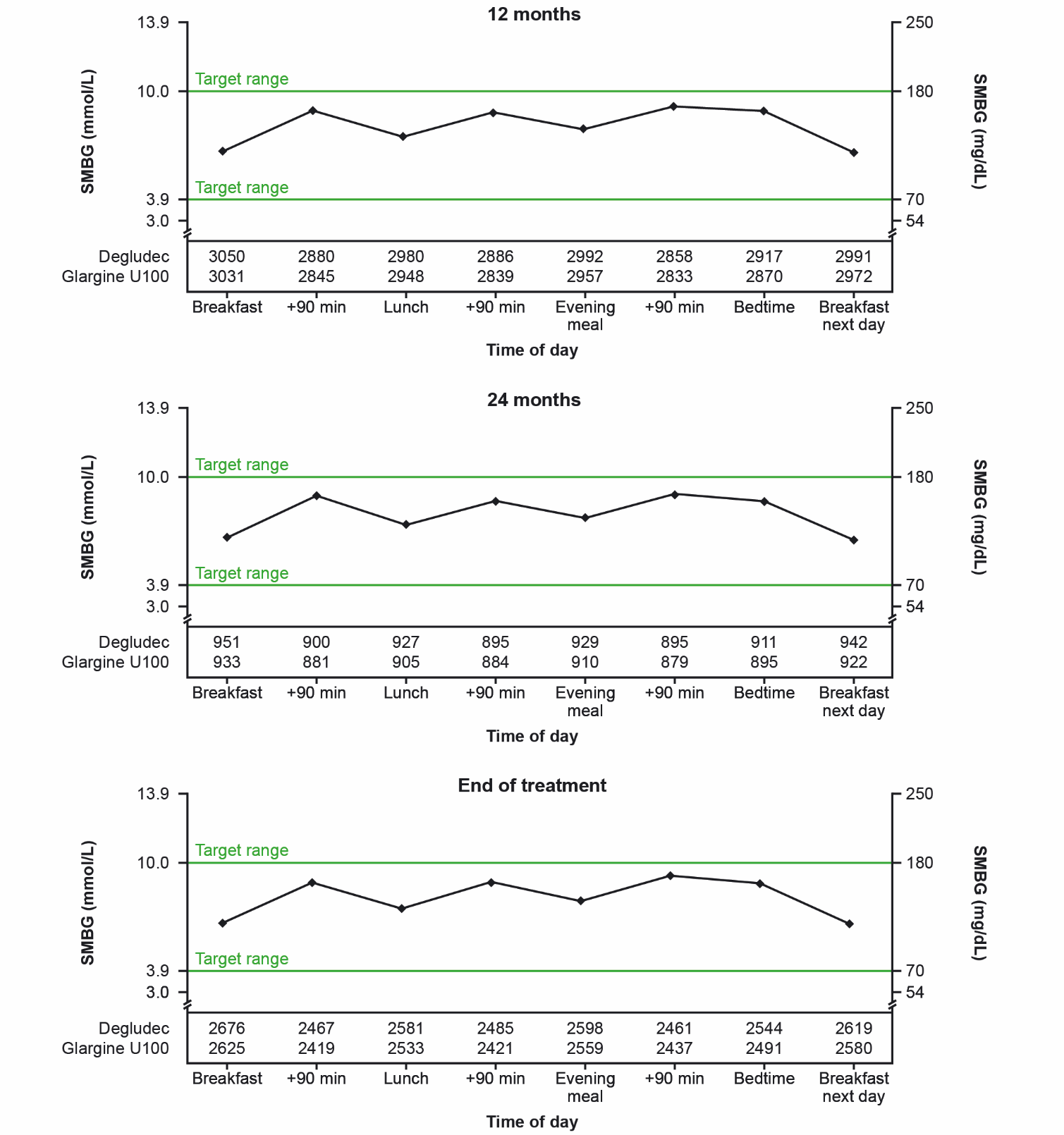


Degludec, insulin degludec (100 units/mL); glargine U100, insulin glargine (100 units/mL); SMBG, self-measured blood glucose.
