# Supplementary material for: Increased Derived Time in Range Is Associated with Reduced Risk of Major Adverse Cardiovascular Events, Severe Hypoglycemia, and Microvascular Events in Type 2 Diabetes: A Post Hoc Analysis of DEVOTE
Source: Diabetes Technol Ther. 2023 May 29;25(6):378–83. doi: 10.1089/dia.2022.0447 (PMC10398723; doi:10.1089/dia.2022.0447)
Supplement: Supplemental data [file Suppl_TableS1.docx]

**Table S1.** Baseline characteristics for participants with complete 8-point SMBG profiles according to their dTIR at 12 months.

| **dTIR (70‒180mg/dL), %** | **<10** | **10‒20** | **20‒30** | **30‒40** | **40‒50** | **50‒60** | **60‒70** | **70‒80** | **80‒90** | **90‒100** |
| --- | --- | --- | --- | --- | --- | --- | --- | --- | --- | --- |
| **N** | 64 | 102 | 224 | 317 | 545 | 63 | 728 | 1056 | 1156 | 1519 |
| **Female, %** | 37.5 | 47.1 | 41.1 | 40.4 | 37.2 | 39.7 | 34.6 | 37.2 | 37.5 | 37.7 |
| **Age, mean years (SD)** | 61.9 (7.1) | 65.7 (6.8) | 66.0 (7.2) | 65.5 (7.5) | 65.7 (7.6) | 66.7 (8.5) | 65.1 (7.2) | 65.5(7.1) | 65.0 (7.0) | 64.0 (6.9) |
| **BMI, mean (SD)** | 34.6 (6.5) | 32.7 (6.7) | 32.7 (6.4) | 32.8 (6.4) | 33.0 (6.8) | 32.1 (5.7) | 33.3 (6.8) | 33.8 (6.5) | 34.0 (7.0) | 33.9 (6.7) |
| **Duration of diabetes, mean years (SD)** | 16.8 (9.5) | 19.1 (9.4) | 17.0 (8.6) | 16.6 (8.9) | 17.0 (8.8) | 16.0 (8.4) | 17.2(9.2) | 17.1(8.7) | 16.3(8.7) | 15.0 (8.4) |
| **HbA1c, %** | 9.6 | 9.4 | 8.9 | 8.8 | 8.7 | 8.8 | 8.5 | 8.3 | 8.1 | 8.1 |

BMI, body mass index; dTIR, derived time in range for combined 8-point SMBG profiles; N, number of participants; SD, standard deviation; SMBG, self-measured blood glucose.
