# Supplementary material for: Increased Derived Time in Range Is Associated with Reduced Risk of Major Adverse Cardiovascular Events, Severe Hypoglycemia, and Microvascular Events in Type 2 Diabetes: A Post Hoc Analysis of DEVOTE
Source: Diabetes Technol Ther. 2023 May 29;25(6):378–83. doi: 10.1089/dia.2022.0447 (PMC10398723; doi:10.1089/dia.2022.0447)
Supplement: Supplemental data [file Suppl_FigureS3.docx]

**Figure S3.** Association between dTIR at 12 months and time to first MACE, severe hypoglycemic episode, or microvascular event.


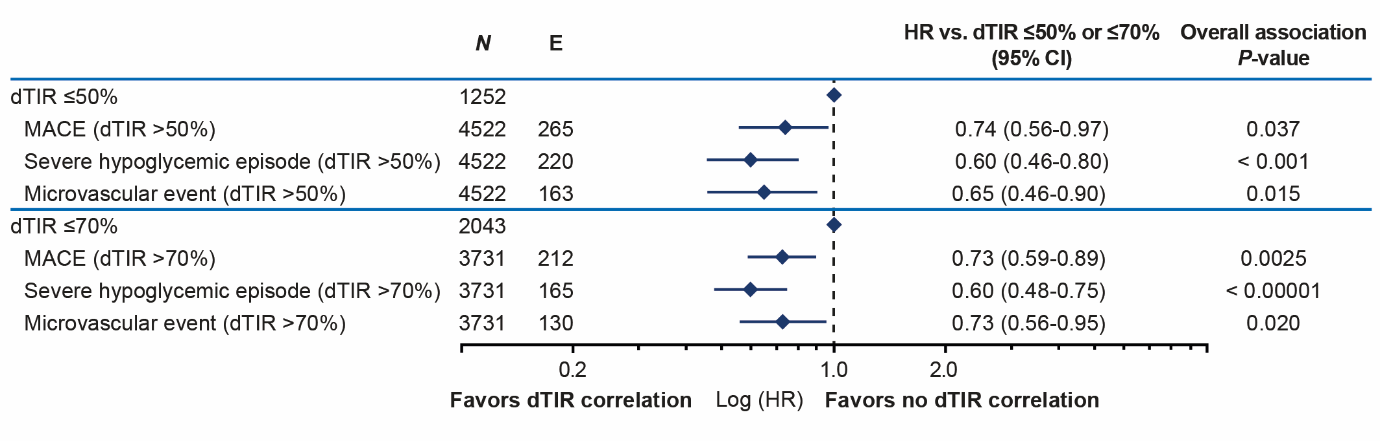


CI, confidence interval; dTIR, derived time in range; E, number of events; HR, hazard ratio; MACE, major adverse cardiovascular event; *N*, number of participants with dTIR.
