# Supplementary material for: Increased Derived Time in Range Is Associated with Reduced Risk of Major Adverse Cardiovascular Events, Severe Hypoglycemia, and Microvascular Events in Type 2 Diabetes: A Post Hoc Analysis of DEVOTE
Source: Diabetes Technol Ther. 2023 May 29;25(6):378–83. doi: 10.1089/dia.2022.0447 (PMC10398723; doi:10.1089/dia.2022.0447)
Supplement: Supplemental data [file Suppl_FigureS4.docx]

**Figure S4.** Association between HbA1c at 12 months, and time to first MACE, severe hypoglycemic episode, or microvascular event on addition of dTIR to the model.


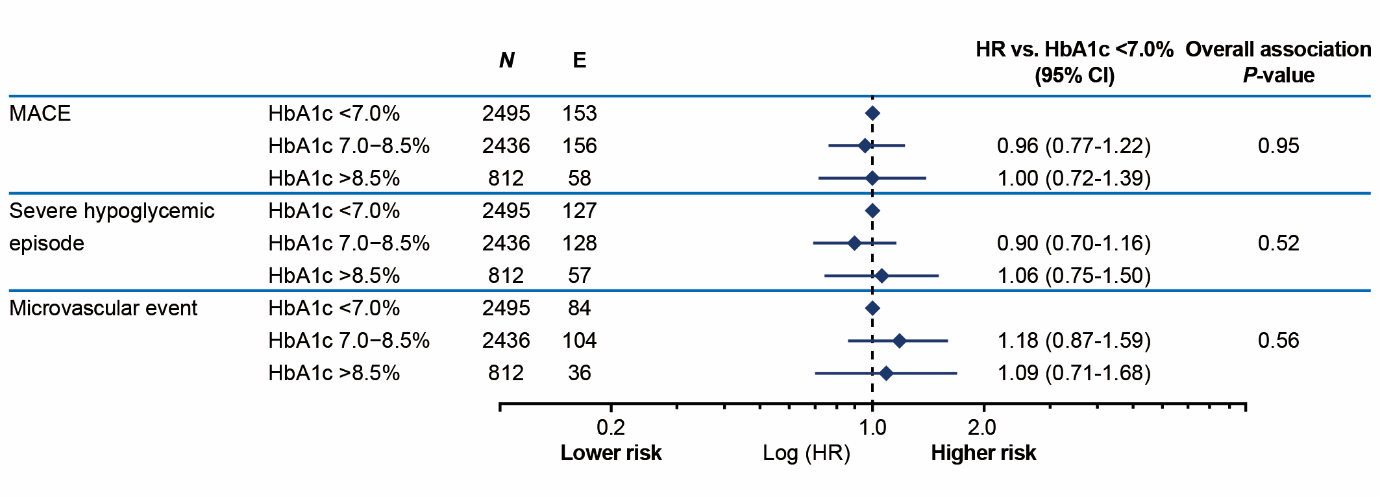


CI, confidence interval; dTIR, derived time in range; E, events; HR, hazard ratio; MACE, major adverse cardiovascular event; *N*, number of participants with dTIR and HbA1c at 12 months.
